# Nonneutralizing antibodies in Nordic persons with moderate hemophilia A and B (the MoHem study)

**DOI:** 10.1016/j.rpth.2024.102611

**Published:** 2024-10-29

**Authors:** Ragnhild J. Måseide, Erik Berntorp, Jan Astermark, Anna Olsson, Maria Bruzelius, Tony Frisk, Vuokko Nummi, Riitta Lassila, Karin Strandberg, Geir E. Tjønnfjord, Pål A. Holme

**Affiliations:** 1 Department of Haematology, Oslo University Hospital, Oslo, Norway; 2 Research Institute of Internal Medicine, Oslo University Hospital, Oslo, Norway; 3 Institute of Clinical Medicine, University of Oslo, Oslo, Norway; 4 Department of Translational Medicine, Lund University, Malmö, Sweden; 5 Department of Haematology, Skåne University Hospital, Malmö, Sweden; 6 Region Västra Götaland, Sahlgrenska University Hospital, Department of Medicine, Gothenburg, Sweden; 7 Department of Medicine Solna, Karolinska Institute, Stockholm, Sweden; 8 Department of Haematology, Karolinska University Hospital, Stockholm, Sweden; 9 Pediatric Coagulation, Karolinska University Hospital, Stockholm, Sweden; 10 Coagulation Disorders Unit, Haematology, Comprehensive Cancer Centre, Helsinki University Hospital and Research Program in Systems Oncology, Faculty of Medicine, Helsinki University, Helsinki, Finland; 11 Department of Clinical Chemistry and Pharmacology, Division of Laboratory Medicine, Coagulation, University and Regional Laboratories Region Skåne, Malmö, Sweden

**Keywords:** arthropathy, hemophilia A, hemophilia B, inhibitors, nonneutralizing antibodies

## Abstract

**Background:**

The impact of nonneutralizing antibodies (NNAs) in moderate hemophilia is elusive.

**Objectives:**

To explore the presence of NNAs in Nordic persons with moderate hemophilia A (MHA) and B (MHB) in relation to treatment modality, clinical outcome, history of inhibitor, and the corresponding factor VIII (FVIII)/factor IX (FIX) gene mutation.

**Methods:**

A cross-sectional multicenter study covering persons with MHA and MHB in Sweden, Finland, and Norway. Inhibitors were analyzed with the Bethesda assay, and NNAs were detected by enzyme-linked immunosorbent assay.

**Results:**

Plasma samples from 137 MoHem study participants (median age 29 years; Q1-Q3, 15-54) were analyzed. NNAs were present in 11 of 82 (13%) of people with MHA and 7 of 55 (13%) of those with MHB irrespective of prophylactic or on-demand treatment, most frequently after 150 exposure days (EDs). Three NNA positive patients had a history of high-titer inhibitor, but current analyses were negative (<0.6 BU/mL). Baseline FVIII/FIX activity was similar among NNA positive and negative patients. Current bleeding rates were low, but patients with NNAs captured a higher Hemophilia Joint Health Score (7 [median]; Q1-Q3, 3-20 vs. 4; 1-9) (*P* = .02) and had more frequently undergone arthroplasty or arthrodesis (5 [33%] vs. 15 [13%]) (*P* = .03).

**Conclusion:**

NNAs were detected in 13% of Nordic persons with MHA and MHB, most frequently after 150 EDs. Patients with NNAs had more severe hemophilic arthropathy than patients without NNAs. The relationship between NNAs and clinical outcome in hemophilia should be further explored in a large cohort including pharmacokinetics and longitudinal observations with repeated blood sampling.

## Essentials

• The impact of nonneutralizing antibodies (NNAs) in moderate hemophilia is elusive.
• NNAs were present in 13% of Nordic persons with moderate hemophilia A and B.
• NNA positivity was associated with lifetime >150 exposure days and more severe arthropathy.
• The clinical consequences of NNAs should be further explored longitudinally in a large cohort.

## Graphical abstract

**Figure:**
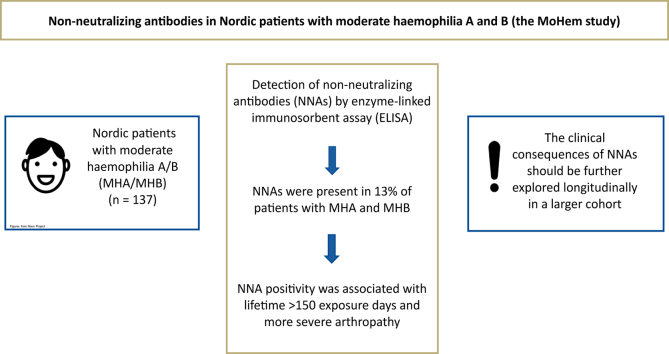

## 1 Introduction

Factor VIII (FVIII) and factor IX (FIX) inhibitors have a major clinical impact in persons with hemophilia A and B as they neutralize replacement therapy with clotting factor concentrates. The clinical consequences of nonneutralizing antibodies (NNAs) have been less studied. In hemophilia A, NNAs against FVIII have been observed throughout all disease severities, with a prevalence between 12% and 54% [[1], [2], [3], [4], [5], [6], [7], [8]]. NNAs have been detected in untreated or minimally treated patients and in 2% to 3% of healthy individuals [[9], [10], [11], [12]]. In severe hemophilia A (SHA), anti-FVIII NNAs were most frequently observed in patients with a history of inhibitor, and the presence of NNAs in patients who were not previously exposed to FVIII concentrates was associated with an increased risk of inhibitor development [6,7]. In hemophilia B, anti-FIX antibodies were found in 40% of patients of different disease severities who tested negative by the Nijmegen-Bethesda assay [13]. However, no NNAs were observed in a Nordic study of persons with severe hemophilia B (SHB) without inhibitors [14].

Inhibitors display higher affinity to FVIII than NNAs found in patients without inhibitors and in healthy individuals [12]. Moreover, NNAs are usually directed toward nonfunctional FVIII epitopes and therefore escape detection in functional assays such as the Bethesda assay but may be detected by an immunologic enzyme-linked immunosorbent assay (ELISA), which identifies all antibodies irrespective of epitope [5]. A possible consequence of NNAs is an increased clearance of administered product, and FVIII-specific NNAs have been associated with shorter FVIII half-life [1,15].

Studies in siblings have supported a genetic predisposition of the FVIII immune response [6]. The causative FVIII gene (*F8*) mutation together with the human leukocyte antigen class II alleles play a pivotal pathophysiological role in inhibitor development [16,17]. In non-SHA, missense mutations affecting the A2 domain of the heavy chain and the A3, C1, and C2 domains of the light chain of the FVIII protein display an increased risk of inhibitor formation [18,19]. The genetic factors associated with NNAs are less characterized.

The MoHem study was conducted to describe and evaluate clinical outcome in persons with moderate hemophilia A (MHA) and B (MHB) as previous publications suggested that these patients were undertreated. The aim of the current study was to explore the presence of NNAs in persons with MHA and MHB in relation to treatment modality, hemophilic arthropathy, history of inhibitor, and the corresponding *F8*/*F9* mutation.

## 2 Methods

The MoHem study was a cross-sectional multicenter study of Nordic persons with MHA and MHB (FVIII/FIX activity [FVIII/FIX:C] 1-5 IU/dL) of all ages affiliated to the Haemophilia Comprehensive Care Centres (HCCC) in Oslo (Norway), Malmö, Gothenburg, Stockholm (Sweden), and Helsinki (Finland). The study received approval from the national ethical committees in all 3 countries and was performed in accordance with the Helsinki Declaration. All participants provided informed consent, with parents providing consent on behalf of their children. Study recruitment was between January 2017 and October 2019, and the main clinical results have been reported previously [20]. MHA and MHB diagnoses were based on lowest historical values of FVIII/FIX:C analyzed at each study center by one-stage or chromogenic assays. However, at enrollment, we collected wash-out samples taken minimum 72 hours after the last administration of clotting factor concentrates. One-stage and chromogenic FVIII/FIX measurements were performed at Skåne University Hospital, Malmö, Sweden as previously described [21].

Inhibitory antibodies were analyzed by the Nijmegen-Bethesda assay with chromogenic FVIII/FIX:C detection and were considered relevant at ≥0.6 Bethesda units per milliliter (BU/mL) in accordance with the recommendations from the Scientific and Standardization Committee of the International Society on Thrombosis and Haemostasis (ISTH) [22,23]. NNAs were detected using an in-house ELISA [6]. Briefly, in the FVIII ELISA-assay, microtiter plates were coated with a mixture of octocog alfa (Takeda Pharma) and turoctocog alfa (Novo Nordisk). In the FIX ELISA, the plates were coated with nonacog alfa (Pfizer). The results of the ELISA were based on optical density of the patient sample compared with that of healthy controls. The cutoff for each test run was determined by analyzing 10 normal plasma samples and calculating the mean +3 standard deviations (SD). Values >3 SD above the mean were defined as positive, and the results were reported semiquantitatively as 3 to 4 or >4 SD above the mean. Both the inhibitor and NNA analyses were performed at Skåne University Hospital, Sweden.

The number of bleeding episodes during the last 12 months as well as the annual factor consumption were self-reported and obtained from clinical records. The definition of ‘joint bleed’ was according to recommendations of the Scientific and Standardization Committee of the ISTH, and bleeds were recorded if they had required clotting factor replacement therapy [23]. Hemophilic arthropathy was evaluated with the Hemophilia Joint Health Score (HJHS) 2.1 and with point-of-care ultrasound according to the Haemophilia Early Arthropathy Detection with Ultrasound (HEAD-US) protocol, which were mainly performed at enrollment [[24], [25], [26]]. We performed subgroup analyses according to the presence of NNAs.

### 2.1 Statistical analyses

Continuous data are summarized as medians and first to third quartiles (Q1-Q3), and categorical data are presented as numbers and percentages. Mann–Whitney U-test was used to compare continuous data, and chi-squared test was used to compare categorical data. A *P* value <.05 was considered statistically significant. The analyses were performed using SPSS Statistics 29.

## 3 Results and Discussion

We analyzed plasma samples from 137 of 145 of the MoHem study participants, representing 62% of all persons with moderate hemophilia attending the 5 Nordic HCCCs. Median age was 29 years (Q1-Q3, 15-54), and 82 (60%) had MHA. The patients not enrolled were older (median 41 years; Q1-Q3, 27-56) (*P* < .01), but the prevalence of MHA (48 [57%]) was similar to that of the study group.

NNAs were detected in 18 participants (13%); 11 of 82 (13%) in persons with MHA and 7 of 55 (13%) in persons with MHB. All participants were previously treated with clotting factor concentrates, and NNAs were most common after a lifetime cumulative number of 150 exposure days (EDs) (Table 1). The majority of the patients (70%) were currently being treated with recombinant products, which were equally distributed between the NNA positive and negative groups. The NNA positive patients included the 3 study participants who had a history of FVIII inhibitor; all were high-titer inhibitors with historical peaks between 34 and 90 BU/mL. Two inhibitor patients had been treated successfully with immune tolerance induction (ITI) or rituximab, and their inhibitor was no longer detectable and considered permanently eradicated. One of them was currently using prophylaxis with recombinant FVIII (moroctocog alfa 4000 IU/kg/y), while the other had not been re-exposed to exogenous FVIII and was treated on-demand with recombinant FVIIa. The third inhibitor patient had failed ITI and was also treated on-demand with recombinant FVIIa. Current inhibitor analyses were negative in all 3 patients, but the patient who failed ITI had a borderline value of 0.5 BU/mL (Table 2). No patients were treated with emicizumab.

**Table 1:** Treatment characteristics and clinical outcome among persons with moderate hemophilia A and B according to the presence of NNAs, excluding the 3 patients with previous FVIII inhibitors.

Parameters	NNA positive (n = 15)	NNA negative (n = 119)	*P*	Total (n = 134)
Age (y)	52 (14-63)	28 (15-50)	.17	29 (15-52)
Caucasian ethnicity	12 (80)	108 (91)	.19	120 (90)
Non-Caucasian ethnicity	3 (20)	11 (9)	.19	14 (10)
Hemophilia A	8 (53)	71 (60)	.64	79 (59)
Hemophilia B	7 (47)	48 (40)	.64	55 (41)
Historical baseline FVIII/FIX:C (IU/dL)	2 (1-4)	2 (2-4)	.93	2 (2-4)
One-stage FVIII/FIX:C (IU/dL)^a^	2.1 (0.8-4.1)	3.3 (1.9-5.4)	.06	3.2 (1.8-5.2)
<2.0 IU/dL	6 (46)	25 (25)		31 (28)
2.1-3.0 IU/dL	2 (15)	20 (20)		22 (20)
3.1-4.0 IU/dL	2 (15)	17 (17)		19 (17)
>4.0 IU/dL	3 (23)	37 (37)		40 (36)
Chromogenic FVIII/FIX:C (IU/dL)^a^	2.1 (0.6-4.3)	2.9 (1.2-4.9)	.24	2.7 (0.9-4.7)
Currently on prophylaxis	7 (47)	47 (40)	.59	54 (40)
Currently treated on-demand	8 (53)	72 (61)	.59	80 (60)
Age at start of prophylaxis (y)	9 (3-22)	10 (4-32)	.48	10 (4-24)
Treated with plasma derived CFC^b^	3 (21)	32 (30)		35 (29)
Treated with recombinant CFC^b^	11 (79)	76 (70)		87 (71)
Lifetime no. of exposure days^c^				
1-50	2 (14)	38 (36)		40 (33)
51-150	0 (0)	11 (10)		11 (9)
>150	12 (86)	58 (54)	.03	70 (58)
Annual factor consumption (IU/kg/y)^d^	1310 (66-5200)	194 (14-2323)	.10	211 (15-2415)
Joint bleeds during the last 12 mo^e^	0 (0-2)	0 (0-1)	.54	0 (0-1)
Other bleeds during the last 12 mo^e^	0 (0-1)	0 (0-1)	.47	0 (0-1)
HJHS total (0-120 points)^f^	7 (3-20)	4 (1-9)	.02	4 (1-10)
HEAD-US total (0-48 points)^g^	2 (0-6)	0 (0-2)	.14	0 (0-2)
Arthroplasties or arthrodeses^e^	5 (33)	15 (13)	.03	20 (15)

Numbers (%) and medians (Q1, first quartile – Q3, third quartile).

CFC, clotting factor concentrates; FVIII/FIX:C, factor VIII/factor IX coagulation activity; HEAD-US, Haemophilia Early Arthropathy Detection with Ultrasound; HJHS, Hemophilia Joint Health Score; NNA, nonneutralizing antibodies.

The number of patients (n) is noted if it deviates from the total number: ^a^n = 13 (NNA+), 99 (NNA−), and 112 (total); ^b^n = 14 (NNA+), 108 (NNA−), and 122 (total); ^c^n = 14 (NNA+), 107 (NNA−), and 121 (total); ^d^n = 14 (NNA+), 115 (NNA−), and 129 (total); ^e^n = 15 (NNA+) and 134 (total); ^f^n = 15 (NNA+), 110 (NNA−), and 125 (total); ^g^n = 10 (NNA+), 91 (NNA−), and 101 (total).

**Table 2:** Individual characteristics of the study participants who were NNA positive.

Patient (age)	Diagnosis	Treatment	ED (d)	History of FVIII/FIX inhibitor	Current inhibitor titer (BU/mL)	NNA (SD above mean)	*F8*/*F9* mutation (HGVS cDNA, HGVS protein)	Exon	FVIII/FIX domain
1 (60 y)	MHA	OD	51-75	Yes	<0.4	>4	c.5374G>T, p.Val1792Leu	15	A3 (LC)
2 (61 y)	MHA	OD	>150	Yes	0.5	>4	c.1207C>G, p.His403Asp	8	A2 (HC)
3 (49 y)	MHA	P	>150	Yes	<0.4	>4	c.1648C>T, p.Arg550Cys	11	A2 (HC)
4 (63 y)	MHA	OD	>150	No	<0.4	3-4	c.5374G>T, p.Val1792Leu	15	A3 (LC)
5 (14 y)	MHA	P	>150	No	<0.4	>4	Intron 22 inv		
6 (24 y)	MHA	P	>150	No	<0.4	3-4	c.217T>C, p.Phe73Leu	2	A1 (HC)
7 (63 y)	MHA	OD	>150	No	<0.4	>4	c.3637delA, p.Ile1213Phefs∗5	14	B (HC)
8 (9 y)	MHA	P	>150	No	<0.4	>4	c.1630G>C, p.hemiz D525H	11	A2 (HC)
9 (21 y)	MHA	P	>150	No	<0.4	>4	c.1017G>A, p.Met339Ile	8	A1 (HC)
10 (64 y)	MHA	P	>150	No	<0.4	>4	c.902G>A, p.Arg301His	7	A1 (HC)
11 (61 y)	MHA	OD	*NA*	No	0.44	>4	c.6978G>A, p.Arg2307Gln	26	C2 (LC)
12 (48 y)	MHB	OD	1-20	No	<0.4	3-4	c.571C>T, p.Arg191Cys	6	Linker (LC)
13 (57 y)	MHB	OD	21-50	No	<0.4	3-4	c.785T>C, p.Ile262Thr	7	Protease (HC)
14 (52 y)	MHB	OD	>150	No	<0.4	3-4	c.1331A>G, p.Tyr444Cys	8	Protease (HC)
15 (13 y)	MHB	P	>150	No	<0.4	>4	c.572G>A, p.Arg191His	6	Linker (LC)
16 (11 y)	MHB	P	>150	No	<0.4	3-4	c.1291T>C, p.Trp431Arg	8	Protease (HC)
17 (78 y)	MHB	OD	>150	No	<0.4	3-4	c.313G>T, p.Gly105Cys	4	EGF1 (LC)
18 (57 y)	MHB	OD	>150	No	<0.4	3-4	c.83G>A, p.Cys28Tyr	1	Signal peptide

NNA optical density values >3 SD above mean of 10 normal plasma samples were defined as positive.

ED, exposure days; EGF1, epidermal growth factor 1; FVIII, factor VIII; FIX, factor IX; *F8*, factor VIII gene; *F9*, factor IX gene; HC, heavy chain; HGVS, Human Genome Variation Society; LC, light chain; MHA, moderate hemophilia A; MHB, moderate hemophilia B; NA, not available; NNA, nonneutralizing antibody; OD, on-demand; P, prophylaxis; SD, standard deviation.

Historical baseline FVIII/FIX:C was similar among NNA positive and negative patients, but at enrollment, there was a trend toward lower one-stage FVIII/FIX:C among the NNA positive patients (Table 1). The numbers of joint bleeds and other bleeds during the last 12 months were zero (median) in both groups. Patients with NNAs captured higher HJHS (7 [median]; Q1-Q3, 3-20 vs. 4; 1-9) (*P* = .02) and had more frequently undergone arthroplasty or arthrodesis (5 [33%] vs. 15 [13%]; *P* = .03) compared with NNA negative, even if the 3 patients with a history of inhibitor were excluded from the analyses (Table 1). The age, the age at start of prophylaxis, current bleeding rates, and the annual factor consumption between NNA positive and negative patients were not distinct (Table 1).

The majority (123/137 [90%]) of the study participants were Caucasians, which included 15/18 (83%) of those with NNAs. Moreover, among the NNA positive patients, 1 was African, 1 Asian, and 1 Asian/Caucasian. As for the overall study population, the underlying *F8*/*F9* mutations were mainly missense mutations located in different gene domains (Table 2).

Accordingly, NNAs were equally prevalent among persons with MHA and MHB. The NNAs occurred less frequently in MHA than previously observed in SHA [6,27]. However, we also detected NNAs in persons with MHB in contrast to a Nordic study of persons with SHB, analyzing NNAs by the same ELISA method in the same laboratory, in which only inhibitor positive patients had NNAs [14]. Moreover, NNAs were found in all 3 study participants who had a history of FVIII inhibitor, as reported in SHA [6].

The presence of NNAs increased with the lifetime cumulative number of EDs, which is in line with previous observations [7]. Patients with NNAs captured higher HJHS scores and had more frequently undergone arthroplasty or arthrodesis than those without detectable NNAs, indicating more severe hemophilic arthropathy. The patients’ age at evaluation as well as their age at start of prophylaxis were similar in both groups. The majority of the patients (60%) were treated on-demand. Thus, the higher number of cumulative EDs among the NNA positive patients may be due to a more severe bleeding phenotype with higher lifetime bleeding rates. Also, previous studies have suggested that NNAs may increase the clearance of FVIII, and FVIII-specific NNAs have been associated with shorter FVIII half-life [1,15]. However, this hypothesis needs to be confirmed with pharmacokinetic data.

As for moderate hemophilia in general, NNA positivity was mainly associated with missense mutations, which were distributed across several domains of the *F8* and *F9* genes. The *F8* mutations c.1207C>G and c.1648C>T (patients 2 and 3, Table 2), both encoding for the A2 domain of the heavy chain of FVIII, have previously been reported in persons with non-SHA and inhibitors [19,28]. The *F8* mutation c.5374G>T in the third inhibitor patient (patient 1, Table 2) is localized in the same gene domain as known inhibitor mutations, encoding for the A3 domain of the light chain of FVIII [19]. Moreover, the intron 22 inversion in patient 5 and the frameshift mutation c.3637delA in patient 7 are both associated with inhibitor development [28]. Intron 22 inversion is commonly seen in SHA; however, the affected patient had been classified as MHA according to the historically lowest available FVIII:C value. Intron 22 inversion has also been described in moderate hemophilia previously [29]. The remaining *F8*/*F9* mutations in the NNA positive patients have not been connected to inhibitors [28]. The *F9* mutation c.572G>A (patient 15) has been observed with an anti-FIX immune response without inhibitor development [13].

### 3.1 Strengths and limitations

Due to a centralized follow-up at the HCCCs and close collaboration between the Nordic countries, we reached a high degree of participation of persons with MHA and MHB. Nevertheless, a general limitation of the study is the low number of participants with NNA antibodies, which hampers the possibility of statistical analyses. The current FVIII/FIX:C, inhibitor, and NNA analyses were centralized to Malmö, Sweden to avoid interlaboratory variation. The ELISA method in our study has been validated and used previously in 2 larger studies [6,30]. It was considered a high-quality assay in the meta-analysis by Abdi et al. [27]. The historical baseline values of FVIII/FIX:C, however, were analyzed at several laboratories at different time points with different methods; thus, classification discrepancies might be considered. Joint health was evaluated with HJHS and Haemophilia Early Arthropathy Detection with Ultrasound, which are reliable and validated tools to detect hemophilic arthropathy [[24], [25], [26]]. Patients with NNAs had more severe arthropathy compared to those without NNAs despite similar ages at study evaluation and at the start of prophylaxis. However, according to the cross-sectional study design with only 1 time point of measuring NNAs without information from repeated blood sampling on development over time, no firm conclusions with respect to the impact of NNAs can be drawn. Furthermore, it would have been valuable with FVIII/FIX pharmacokinetics to assess the impact of NNAs on FVIII/FIX clearance [1,15].

## 4 Conclusions

NNAs were detected in 13% of Nordic persons with MHA and MHB, most frequently after 150 EDs. Current bleeding rates were low and did not differ between NNA positive and negative patients. However, patients with NNAs captured higher HJHS scores and had more frequently undergone arthroplasty or arthrodesis. The higher number of cumulative EDs among the NNA positive patients may be due to a more severe bleeding phenotype with higher lifetime bleeding rates. However, the relationship between NNAs and clinical outcome in hemophilia should be further explored in a large cohort including pharmacokinetics and the presence of NNAs over time.
